# Next-Generation Sequencing: A Review of Its Transformative Impact on Cancer Diagnosis, Treatment, and Resistance Management

**DOI:** 10.3390/diagnostics15192425

**Published:** 2025-09-23

**Authors:** Alexandru Isaic, Nadica Motofelea, Teodora Hoinoiu, Alexandru Catalin Motofelea, Ioan Cristian Leancu, Emanuela Stan, Simona R. Gheorghe, Alina Gabriela Dutu, Andreea Crintea

**Affiliations:** 1 Department of General Surgery, “Victor Babes” University of Medicine and Pharmacy, 300041 Timisoara, Romania; isaic.alexandru@umft.ro; 2 Department of Obstetrics and Gynecology, “Victor Babes” University of Medicine and Pharmacy, 300041 Timisoara, Romania; 3 Department of Clinical Practical Skills, “Victor Babes” University of Medicine and Pharmacy, 300041 Timisoara, Romania; tstoichitoiu@umft.ro; 4 Center for Advanced Research in Cardiovascular Pathology and Hemostaseology, “Victor Babes” University of Medicine and Pharmacy, 300041 Timisoara, Romania; 5 Centre for Molecular Research in Nephrology and Vascular Disease/MOL-NEPHRO-VASC, “Victor Babes” University of Medicine and Pharmacy, 300041 Timisoara, Romania; alexandru.motofelea@umft.ro; 6 Department of Computer and Information Sciences, Discipline Artificial Intelligence, University of Anglia Ruskin, Cambridge CB1 1PT, UK; icl103@student.aru.ac.uk; 7 Department of Neuroscience, Discipline of Forensic Medicine, Bioethics, Deontology and Medical Law, “Victor Babes” University of Medicine and Pharmacy, 300041 Timisoara, Romania; emanuela.stan@umft.ro; 8 Department of Molecular Sciences, Iuliu Hațieganu University of Medicine and Pharmacy, 400349 Cluj-Napoca, Romania; gheorghe.simona@umfcluj.ro (S.R.G.); alina.dutu@umfcluj.ro (A.G.D.); crintea.andreea@umfcluj.ro (A.C.)

**Keywords:** Next-Generation Sequencing, precision oncology, liquid biopsy, comprehensive genomic profiling, multi-omics, spatial transcriptomics, cancer biomarkers, targeted therapy

## Abstract

**Background/Objectives**: Next-Generation Sequencing (NGS) has transformed cancer diagnostics and treatment by enabling comprehensive genomic profiling of tumors. This review aims to summarize the current applications of NGS in oncology, highlighting its role in early detection, precision therapy, and disease monitoring. **Methods**: We conducted a comprehensive review of the recent literature, focusing on the application of NGS in cancer care. **Results**: NGS enables high-resolution genomic profiling, identifying actionable mutations (e.g., EGFR, KRAS, and ALK) and immunotherapy biomarkers (e.g., PD-L1, TMB, and MSI), guiding personalized treatment selection and improving outcomes in advanced malignancies. Liquid biopsy enhances diagnostic accessibility and enables real-time monitoring of minimal residual disease and treatment resistance. Despite these advances, widespread clinical adoption remains constrained by technical limitations (e.g., coverage uniformity and sample quality), economic challenges (high costs and complex reimbursement), and interpretative issues, including the management of variants of uncertain significance (VUSs). **Conclusions**: NGS is central to precision oncology, enabling molecularly driven cancer care. Integration with artificial intelligence, single-cell sequencing, spatial transcriptomics, multi-omics, and nanotechnology promises to overcome current limitations, advancing personalized treatment strategies. Standardization of workflows, cost reduction, and improved bioinformatics expertise are critical for its full clinical integration.

## 1. Introduction

Cancer, a complex disease characterized by profound genetic alterations and cellular dysregulation, remains a major global health challenge and is currently the second leading cause of death worldwide [1,2]. In 2022, there were an estimated 20 million new cancer cases and 9.7 million deaths, with more than 53 million individuals living within five years of a cancer diagnosis [3]. Approximately one in five people will develop cancer during their lifetime, while about one in nine men and one in twelve women will ultimately die from the disease [2]. The global cancer burden is further shaped by striking inequities in access to care and survival outcomes, particularly between countries at different Human Development Index (HDI) levels [1,3,4,5]. For example, women in low-HDI regions are nearly 50% less likely to be diagnosed with breast cancer compared to those in high-HDI countries, yet they face substantially higher mortality risks [5,6,7], largely due to delayed diagnosis and limited access to effective treatment [8,9]. These disparities highlight the urgent global need for advanced and equitable cancer diagnostics and therapies.

Next-Generation Sequencing (NGS) has emerged as one of the most transformative technologies in oncology. By enabling comprehensive genomic, transcriptomic, and epigenomic profiling, NGS facilitates the identification of driver mutations, fusion genes, and predictive biomarkers across diverse cancer types. Its applications extend well beyond single-disease contexts, spanning hematological malignancies and solid tumors, and underpinning the paradigm shift toward precision oncology. For instance, lung cancer—the leading cause of cancer mortality worldwide, with 2.5 million new cases and 1.8 million deaths in 2022 alone [1,2,10,11,12]—illustrates how molecular profiling can refine classification (e.g., NSCLC versus SCLC [11,12,13,14,15,16,17,18]) and improve treatment strategies. Similar advances are seen in breast, colorectal, and hematologic cancers, where NGS-based molecular characterization has guided the adoption of more effective, personalized therapies [16,17,18,19,20,21,22,23].

Traditional cancer treatments such as surgery, chemotherapy, and radiotherapy have saved lives but are limited by toxicities and reduced efficacy in advanced disease [4,15,16,17,18,19,20,21,22,23,24,25]. These challenges have driven the development of genomic-informed, multi-agent, and pathway-targeted strategies that exploit tumor-specific molecular alterations [26,27]. By focusing on the genetic and molecular basis of cancer, precision therapies informed by NGS aim to improve treatment outcomes while minimizing collateral toxicity [28,29,30,31,32].

In this review, we synthesize current evidence on the role of NGS in advancing cancer diagnosis, treatment selection, and disease monitoring, with an emphasis on its integration into precision oncology. We outline the technological principles of NGS, compare the strengths and limitations of various sequencing platforms, and highlight their clinical applications, including comprehensive genomic profiling, liquid biopsy, and resistance monitoring. In this review, the term NGS is used in its broader sense, referring to both second-generation (short-read) and third-generation (long-read) sequencing technologies, as both are increasingly relevant for cancer research and clinical applications. Additionally, we explore emerging biomarkers for immunotherapy response prediction and discuss challenges in bringing NGS into routine clinical practice. Our goal is to provide clinicians and researchers with a comprehensive overview of how NGS is transforming cancer care across multiple tumor types.

## 2. NGS: Principles, Technologies, and Workflow

### 2.1. Fundamental Principles and Evolution of NGS

NGS has transformed molecular biology by redefining approaches to disease research and clinical diagnostics. Since its widespread adoption around 2008, NGS has progressively displaced traditional Sanger sequencing, becoming integral to contemporary genomic medicine, particularly in oncology [33,34,35,36,37]. Second-generation platforms (e.g., Illumina, Roche/454, ABI/SOLiD) and third-generation technologies (e.g., PacBio (Pacific Biosciences of California, Inc., Menlo Park, CA, USA.) single-molecule real-time sequencing, Oxford Nanopore) represent a major advance in sequencing throughput, read length, and analytical resolution compared to earlier methods [37,38,39]. NGS enables comprehensive interrogation of genomes with the capacity to detect point mutations, copy number variations, and novel structural alterations, including chromosomal rearrangements, which are critical in understanding disease mechanisms [35,36,37,40]. In oncology, it provides clinically actionable molecular insights, guiding diagnosis, prognostication, therapeutic selection, and monitoring of treatment response [41,42,43]. A defining attribute of NGS is its massively parallel sequencing architecture, enabling the concurrent analysis of millions of DNA fragments. This allows simultaneous evaluation of hundreds to thousands of genes in a single assay, offering a comprehensive genomic landscape rather than the fragmented approach inherent to Sanger sequencing [35,44]. Such multi-gene, high-throughput capacity is essential for complex diseases like cancer, which are driven by diverse and interacting genomic alterations. By facilitating integrated profiling, NGS has deepened our understanding of tumor pathogenesis and improved the precision of both diagnostic and therapeutic strategies [42,45].

### 2.2. NGS Technologies: Comparison with Sanger and PCR

NGS platforms differ markedly in throughput, read length, error profiles, and analytical scope, making the selection of an appropriate technology critical for clinical and research applications [46,47]. Like Sanger sequencing, NGS relies on DNA polymerase incorporating fluorescently labeled nucleotides into a growing DNA template. However, the key distinction lies in throughput: Sanger sequencing processes one DNA fragment at a time, making it laborious, costly, and time-consuming for large-scale analysis [46]. It also exhibits lower sensitivity, with a detection limit typically around 15–20%, and is not cost-effective for analyzing more than 20 targets [48,49]. While Sanger sequencing offers a familiar workflow and can sequence up to 1000 base pairs, its limited throughput and scalability make it less suitable for comprehensive genomic analyses [50]. Consequently, Sanger sequencing is now primarily utilized for validating results initially identified by NGS [37].

In contrast, NGS employs massively parallel sequencing, simultaneously analyzing millions of DNA fragments in a single run, which enables the interrogation of hundreds to thousands of genes concurrently [36]. This parallel architecture provides markedly increased sequencing depth and sensitivity, detecting low-frequency variants down to ~1% variant allele frequency, and shortens turnaround times—an entire human genome can be sequenced in approximately one week, compared with years using Sanger technology [36,51]. NGS also delivers superior discovery power, detecting novel or rare variants, structural rearrangements, and large chromosomal abnormalities at single-nucleotide resolution [35,52]. This comprehensive genomic coverage and higher capacity with sample multiplexing make NGS a more cost-effective solution for screening a larger number of samples and reliably detecting genes associated with tumor formation and progression [53].

When compared to polymerase chain reaction (PCR), which targets predefined loci and is cumbersome for multiplexed analyses, NGS provides single-base resolution and can simultaneously identify single-nucleotide polymorphisms (SNPs), insertions/deletions (indels), copy number variations (CNVs), and structural variants [36,51]. This increased throughput and comprehensive analysis capability make NGS a superior choice for complex genomic profiling (Table 1).

## 3. Major NGS Platforms: Illumina, Oxford Nanopore, and Pacific Biosciences (PacBio)

The choice of NGS platform is a strategic decision, directly influencing the feasibility and success of a research or clinical project, as each platform excels in different areas.

Illumina sequencing dominates second-generation NGS due to its exceptionally high throughput, low error rates (typically 0.1–0.6%), and attractive cost per base [36,54]. It uses sequencing-by-synthesis chemistry, enabling millions of DNA fragments to be sequenced in parallel on a flow cell [46,55]. Short reads (75–300 bp) provide high coverage and precision, suitable for genome resequencing, transcriptome profiling, and variant calling. Established bioinformatics pipelines (e.g., BWA, GATK, STAR) are well-optimized for Illumina data [35,54].

Oxford Nanopore Technologies (ONT) has introduced a distinctive approach with its nanopore sequencing, which involves directly reading single DNA molecules as they traverse a protein nanopore [56]. A key advantage of ONT is its ability to produce ultra-long reads, frequently exceeding tens of thousands of base pairs and even reaching over 100,000 base pairs with specialized protocols [52,57]. These extended read lengths are particularly valuable for de novo genome assembly, comprehensive structural variant detection, and haplotype phasing, areas where short reads often fall short [56]. Furthermore, nanopore sequencing facilitates real-time data acquisition and analysis, making it suitable for rapid diagnostics and field-based applications. While traditionally less accurate than Illumina, with error rates around 10–15%, continuous advancements in base-calling algorithms and chemistry, such as Q20+ chemistry and duplex reads, have significantly narrowed this accuracy gap [58,59]. Bioinformatics analysis for ONT data can be more complex due to higher variability, but long-read tools like minimap2, Flye, Shasta, and polishing tools like Medaka or Homopolish are now widely used [60].

Pacific Biosciences (PacBio) is renowned for its single-molecule real-time (SMRT) sequencing technology, which also generates long reads, capable of reaching lengths of up to 100,000 base pairs [61]. PacBio sequencing provides a comprehensive view of complex genomic regions, including those with high GC content or repetitive sequences. The platform’s high accuracy, particularly with its newer HiFi reads (achieved through circular consensus sequencing, where the same molecule is sequenced multiple times to build a consensus), combines the benefits of long reads with high precision, with accuracy close to Illumina’s [61,62]. This makes PacBio excellent for applications such as full-length transcript sequencing, metagenomics, and epigenomics [63,64]. The primary drawbacks of PacBio sequencing are its relatively lower throughput and higher cost per base compared to Illumina, which can be a limiting factor for very large-scale projects, although newer systems like Revio are bringing costs down for whole-genome or large transcriptome projects [65]. Similar to ONT, PacBio data initially require more advanced bioinformatics handling, though tools like Hifiasm have streamlined HiFi data analysis [66].

The diversity among these NGS platforms underscores the principle that no single technology is universally superior; the optimal choice depends on project-specific requirements. For instance, high-throughput variant calling in known genomic regions often favors Illumina due to its cost-effectiveness and accuracy. In addition to Illumina short-read sequencing, newer platforms such as Pacific Biosciences (PacBio) and MGI are increasingly relevant, although less frequently used in cancer research. PacBio’s long-read technology enables accurate detection of structural variations, full-length isoforms, and complex splicing or fusion events, providing valuable insights into tumor heterogeneity and evolution [67]. MGI platforms, meanwhile, offer rapid and cost-efficient high-throughput sequencing, supporting large-scale genomic profiling and biomarker discovery in the context of therapy response [68,69]. Both technologies also facilitate multi-omics approaches that integrate genomic and transcriptomic data, helping to link genetic alterations with immune microenvironment dynamics and advancing precision oncology [70]. This tailored selection maximizes resource utilization and enhances the likelihood of generating clinically or scientifically relevant insights. Table 2 compares Illumina, ONT, and PacBio sequencing platforms across key technical and practical metrics.

### 3.1. Types of NGS: Whole-Genome, Whole-Exome, and Whole-Transcriptome Sequencing

NGS encompasses various approaches, each designed to target specific components of the genome or transcriptome, depending on the research or clinical objective. The primary types include Whole-Genome Sequencing (WGS), Whole-Exome Sequencing (WES), and Whole-Transcriptome Sequencing (RNA-Seq) [35]. Next-generation sequencing (NGS) has reshaped cancer genomics by enabling high-resolution profiling of genetic alterations in tumor tissues. Within the spectrum of NGS approaches, Whole-Genome Sequencing (WGS) and Whole-Exome Sequencing (WES) occupy complementary roles in identifying key classes of mutations, including single-nucleotide variants (SNVs), small insertions and deletions (indels), copy-number variations (CNVs), and structural variations (SVs).

WGS surveys the entire genome, capturing both coding and non-coding regions where alterations relevant to oncogenesis may occur. This broad scope allows for the detection of variants that might be missed by more targeted methods. By contrast, WES focuses on the ~1.5% of the genome that encodes proteins, offering a cost-effective and efficient means of interrogating the regions most frequently implicated in cancer [71]. However, because of its restricted coverage, WES is less suited to detecting regulatory mutations or large-scale structural rearrangements that can strongly influence gene expression and tumor development [72]

The accuracy of variant detection also depends on the sequencing platform and read length. Short-read technologies, such as Illumina, are highly effective for identifying SNVs and small indels due to their high throughput, lower cost, and strong performance in pinpointing point mutations. As such, they remain the mainstay for initial mutational screening in clinical tumor diagnostics. Their limitations, however, become apparent when attempting to resolve larger or more complex genomic alterations. The fragmented nature of short reads often makes it difficult to reconstruct structural variations, although advances in bioinformatics pipelines and variant-calling algorithms have improved performance in this area [73].

By contrast, long-read sequencing technologies, including platforms developed by (PacBio) and Oxford Nanopore, are particularly well suited for detecting SVs and large genomic rearrangements. Their ability to generate continuous long reads allows for more complete assemblies of complex regions and facilitates the identification of events such as gene fusions, insertions, and large deletions—alterations that are often missed with short-read sequencing [74]. Moreover, the additional genomic context provided by long reads helps to clarify how structural changes affect gene regulation and tumor biology.

Although long-read sequencing offers significant advantages for detecting SVs and CNVs, it typically involves higher costs and lower throughput compared to short-read approaches. For this reason, integrative strategies that combine both technologies are increasingly being adopted, as they provide a more comprehensive view of the tumor genome and enable the detection of a wider range of mutation types [75].

#### 3.1.1. Whole-Genome Sequencing (WGS)

WGS involves sequencing the entire genome, providing the most comprehensive genetic information available. This approach enables a thorough characterization of an individual’s complete genetic blueprint, facilitating the identification of various factors that influence disease occurrence and evolution [35,76]. It captures SNVs, small insertions/deletions (indels), CNVs, structural rearrangements, and other complex genomic features that drive cancer [77]. Despite its comprehensiveness, WGS presents certain limitations. It is significantly more expensive than WES, with costs potentially two to three times higher, and in some comparisons, up to five times higher for maximum costs [77,78,79,80]. WGS generates an immense volume of data, approximately 120 gigabytes (Gb) per patient compared to 10 Gb for WES, necessitating substantially more resources for data storage, computing power, and analysis time [77]. Furthermore, while WGS aims for complete coverage, it has a limited ability to reliably detect certain types of variants for current clinical use. These include insertions, deletions, copy number variations, long repeat sequences, triplet repeat expansions, structural chromosomal rearrangements, polyploidy, mosaicism, and variants located in regions with high sequence identity, such as paralogous genes and pseudogenes [81]. Interpreting variants in non-coding regions—which represent over 98% of the genome—also poses major challenges due to limited functional annotation.

#### 3.1.2. Whole-Exome Sequencing (WES)

WES focuses specifically on sequencing the coding regions of genes, known as the exome, which constitutes approximately 1% of the human genome [76,82]. This targeted approach is based on the premise that the majority of known disease-causing genetic variants reside within these protein-coding regions [83]. Consequently, WES is unable to detect changes that occur in regulatory or promoter regions, or other non-coding areas of the genome, with the exception of canonical splicing regions immediately adjacent to coding sequences [84]. WES is particularly powerful in identifying the genetic causes of WES and offers several advantages, primarily being faster and more cost-effective than WGS [81]. It also tends to generate fewer variants of uncertain significance compared to WGS, simplifying the interpretation process [85]. WES is particularly powerful in identifying the genetic causes of rare diseases, especially when applied in a trio exome sequencing format (sequencing the affected individual and both parents) [84,86]. However, WES also has distinct limitations. Its inability to detect variants in non-coding regions means that potentially important regulatory mutations or structural changes outside the exome are missed. While it can detect single-nucleotide variants and small insertions/deletions, its reliability for detecting CNVs is lower than WGS, and it generally does not detect large structural rearrangements [87,88]. Mosaicism, where a genetic alteration is present in only a subset of cells, may also not be reliably detected due to limitations in read depth [87]. Technical challenges persist in sequencing certain genomic regions, such as those containing pseudogenes or repetitive elements. Furthermore, despite generating fewer VUS than WGS, the clinical interpretation of the large number of identified variants can still be challenging, and there is an increased risk of incidental findings—the discovery of genetic information unrelated to the primary reason for testing [89].

#### 3.1.3. Whole-Transcriptome Sequencing (RNA-Seq)

RNA-Seq involves the sequencing of RNA transcripts to analyze gene expression, identify transcript variants, and study splicing events [90,91]. This technique is particularly valuable when there is a specific interest in understanding the activity of a set of genes, pathways, or regulatory elements, and it can be instrumental in identifying new biomarkers, gene variants, or mutations. However, RNA-Seq presents significant technical challenges. RNA quality and integrity are pivotal—RNA is prone to degradation, and degraded input (e.g., low DV200) significantly undermines reproducibility and gene expression accuracy [92,93,94,95]. Library preparation introduces biases during cDNA synthesis, fragmentation, and PCR amplification, affecting transcript representation [96,97]. Compounding these challenges is the immense complexity of RNA-Seq datasets, which require sophisticated computational tools for normalization, differential expression analysis, and transcript-level profiling [98]. Batch effects, variable sample quality, and inconsistent protocols across laboratories further threaten reproducibility and inter-study comparability [99,100].

Moreover, conventional RNA-Seq typically fails to capture the full spectrum of post-transcriptional RNA modifications and non-polyadenylated transcripts (e.g., histone mRNAs), limiting insights into regulatory layers beyond gene expression [101]. Removal of highly abundant ribosomal RNA (rRNA) and messenger RNA (mRNA) enrichment steps adds complexity and potential bias, and RNA-Seq is inefficient when only a few target transcripts are relevant, generating excessive unused data volume.

In the context of cancer research, selecting the appropriate sequencing strategy requires balancing genomic coverage, cost, data interpretability, and intended biological insight. Although RNA-Seq provides dynamic expression-level information, its utility must be weighed against practical limitations, especially when the disease question focuses on known genetic variants rather than expression profiles.

Traditional statistical methods, combined with machine learning and AI, are increasingly applied to RNA-Seq data to build predictive scoring systems for prognosis and therapy response, particularly for immune checkpoint inhibitors (ICIs) such as PD-1/PD-L1 therapies [102,103]. A recent advance in this direction is NetBio, a machine learning framework that applies network-based analysis of transcriptomic data from more than 700 ICI-treated patients. NetBio accurately predicted responses across melanoma, gastric cancer, and bladder cancer, and outperformed conventional biomarkers such as PD-L1 expression or tumor microenvironment signatures [104]. Similarly, in melanoma, the iDICss model integrated genomic and transcriptomic data via independent component analysis to define immune-driven axes of variation. This approach stratified patients into high- and low-risk groups with strong predictive power for survival and immunotherapy benefit, while also correlating with drug sensitivity [105].

Beyond these integrative models, RNA-Seq also helps uncover survival-associated biomarkers in individual cancers. For example, in breast cancer, the MIRS (metastatic and immunogenomic risk score) combines tumor-infiltrating immune cell (TIIC) profiles with metastatic features using a neural network model. Patients classified as MIRShigh had worse survival outcomes but were more responsive to chemotherapy, while MIRSlow patients had better overall survival and greater sensitivity to immunotherapy [106]. In ovarian cancer, early transcriptomic studies identified MAGP2 as a poor-prognosis marker in advanced papillary serous tumors. Functional analyses revealed that MAGP2 promotes tumor cell survival and angiogenesis through αVβ3 integrin signaling, highlighting its role as both a prognostic biomarker and a potential therapeutic target [107].

RNA-Seq also captures more subtle regulatory changes that shape therapy response. In melanoma, investigators demonstrated that shifts in 3′-UTR alternative polyadenylation (APA) distinguished responders from non-responders to ICI therapy, linking APA usage with immune signaling, angiogenesis, and DNA repair pathways. They developed an APA-based scoring system (IRAPAss) that successfully predicted ICI response across multiple patient cohorts, with validation at the single-cell level revealing differences in APA usage across T-cell subsets [108].

While these studies illustrate the promise of RNA-Seq, they also highlight a challenge: the lack of reproducibility across datasets and cancer types. A large benchmarking effort systematically evaluated nearly 50 transcriptomic biomarker signatures across 29 ICI-treated cohorts, demonstrating that many predictors fail to generalize beyond their original study context. However, some scores, such as TIDE, CYT, PASS-ON, and EIGS_ssGSEA, consistently correlated with outcomes. This effort led to the development of ICB-Portal, a public resource that allows for standardized evaluation of new transcriptomic biomarkers in immunotherapy [109].

Together, these advances show how RNA-Seq, when combined with advanced computational methods, can reveal novel biomarkers, predict treatment response, and uncover resistance mechanisms across cancer types. From immune network-based predictors (NetBio, iDICss) to risk scores integrating immune and metastatic features (MIRS), angiogenic drivers (MAGP2), and APA-based stratification (IRAPAss), transcriptomic analysis is increasingly central to precision oncology. Moving forward, emphasis will likely be placed on validating these models across large, diverse cohorts, integrating them with multi-omics and single-cell data, and translating them into clinically usable pipelines. Table 3 compares WGS, WES, and RNA-Seq across target regions, applications, advantages, and limitations.

### 3.2. The NGS Workflow: From Sample to Insight

NGS has revolutionized molecular oncology, but its clinical and research utility depends on a meticulously coordinated workflow comprising three interdependent phases: sample preparation, sequencing, and bioinformatics analysis. Each phase is critical; errors introduced early can propagate downstream, affecting variant detection and interpretation [110,111].

#### 3.2.1. Sample Preparation

The initial and often most critical step in the NGS workflow is sample preparation. This involves the isolation of nucleic acids, either DNA or RNA, from the biological sample. The process begins with lysing cells to release their genetic material, followed by purification to separate the nucleic acids from other cellular components, yielding pure DNA or RNA ready for downstream processing [111,112]. A crucial component of sample preparation is library preparation, where the isolated DNA or RNA samples are fragmented into smaller, manageable pieces. Subsequently, specialized adapter sequences are ligated to the ends of these fragments [110]. This adapter ligation is essential for preparing the samples for efficient amplification and subsequent sequencing on NGS instruments. Significant challenges can arise during sample preparation, particularly concerning sample quality. Degraded or contaminated samples, frequently encountered with formalin-fixed paraffin-embedded (FFPE) tissues, can severely compromise library quality and lead to sequencing failures [113]. RNA, in particular, is highly susceptible to degradation, necessitating meticulous handling to prevent RNase contamination [112]. To mitigate these issues, careful handling, optimization of fragmentation time, and increasing DNA input for low-quality samples are recommended. For RNA sequencing, enrichment steps are often crucial [112]. mRNA constitutes only a small fraction (about 5%) of total cellular RNA, while rRNA is highly abundant [114]. Therefore, workflows typically include steps to enrich for transcripts, such as selecting for the poly(A) tail found on eukaryotic mRNA, or to deplete highly abundant RNAs like rRNA, especially when dealing with degraded or low-quality RNA samples [115,116]. Maintaining clean workspaces, strictly following established protocols, incorporating positive and negative controls, and optimizing input nucleic acid amounts are vital practices to ensure successful library construction and minimize biases [96,112].

#### 3.2.2. Sequencing

Prepared libraries are then loaded onto sequencing platforms. Illumina’s sequencing-by-synthesis (SBS) technology dominates clinical NGS, offering short, highly accurate reads (error rates 0.1–0.6%) suitable for genome resequencing, exome analysis, and transcriptomics [36,117]. Oxford Nanopore and Pacific Biosciences (PacBio) provide long-read technologies, enabling resolution of structural variants, repetitive regions, and haplotypes, though at higher cost or with trade-offs in raw accuracy [36,52,61]. The choice of platform depends on the project’s goals: short reads for high-throughput profiling versus long reads for complex genome architecture.

#### 3.2.3. Bioinformatics Analysis

The bioinformatics analysis phase is arguably the most critical for transforming the raw sequencing data into meaningful biological and clinical insights [118]. Without robust bioinformatics pipelines, the immense data output from NGS would be essentially unusable. This complex process typically involves several sequential steps:

- Data cleanup: The initial step involves filtering raw sequencing reads to remove low-quality data, adapter sequences, and other artifacts that could interfere with downstream analysis [119]. While sequencing instruments often perform initial cleanup, third-party tools like FastQC can be used for further quality assessment [120,121].
- Alignment/mapping: The cleaned reads are then aligned or mapped to a known reference human genome (e.g., GRCh38) using sophisticated algorithms implemented in software such as BWA or Bowtie2 [122,123]. This process determines the precise genomic location from which each read originated, effectively reconstructing the sequenced genome or transcriptome. Accurate alignment is paramount for reliable variant calling in subsequent steps [124].
- PCR duplicate removal: During library preparation, PCR amplification can lead to multiple identical copies of the same DNA fragment. These PCR duplicates are identified and removed to prevent them from skewing variant calling statistics, ensuring that each original template molecule is counted only once [120].
- Variant calling: This stage involves comparing the aligned read sequences to the reference sequence to identify locations where they differ. Sophisticated algorithms, often implemented in tools like GATK, FreeBayes, and SAMtools, analyze these differences to detect various types of genetic variations. These include SNPs, small insertions and deletions (INDELs), larger structural variations (SVs), and CNVs [125]. The selection of the appropriate variant caller is crucial, as different tools may perform better with specific data types or sequencing technologies [126,127].
- Variant annotation: Raw variants are functionally annotated using tools such as ANNOVAR or Ensembl VEP, integrating information on gene context, predicted functional impact, population frequencies, conservation scores, and known disease associations from curated databases (e.g., ClinVar, COSMIC) [128,129].
- Variant interpretation: This step represents the greatest challenge. Interpretation involves assessing the pathogenicity and clinical significance of detected variants, particularly in the context of VUS or incidental findings. Expert judgment is required to integrate molecular data with clinical context, guided by frameworks such as the ACMG/AMP variant classification guidelines [119,130]. Ethical considerations are especially critical for managing incidental findings unrelated to the primary clinical question [89].

The quality and success of each step, from the initial sample preparation to the final bioinformatics analysis, critically impact the downstream data interpretation. The most significant bottleneck, however, lies in the complex and expert-dependent interpretation of the vast genomic data, particularly concerning variants of uncertain significance and incidental findings [89,131]. This limits the scalability of NGS adoption and highlights the ongoing need for advanced bioinformatics tools and standardized interpretation guidelines. The human element of interpretation, requiring specialized expertise, remains a critical, rate-limiting step in fully leveraging the power of NGS, necessitating the development of robust bioinformatics solutions and potentially artificial intelligence-driven approaches to streamline this process [37,132,133].

## 4. Applications of NGS in Modern Cancer Management

NGS has profoundly transformed modern cancer management, revolutionizing how clinicians diagnose, stratify, and monitor malignancies. By enabling high-resolution genomic profiling, NGS supports precision oncology approaches that align treatment with the molecular features of each patient’s tumor, while providing tools for dynamic disease surveillance and resistance tracking [35,43,47].

### 4.1. Revolutionizing Cancer Diagnostics: Biomarker Discovery and Comprehensive Genomic Profiling (CGP)

NGS has fundamentally altered cancer diagnostics by enabling comprehensive genomic profiling (CGP), an integrated approach that detects a broad spectrum of clinically actionable alterations and emerging composite biomarkers [34,134,135,136]. This multi-dimensional profiling identifies key drivers of oncogenesis, offering a holistic understanding of tumor biology and improving patient stratification for tailored treatments.

#### 4.1.1. Liquid Biopsy: A Non-Invasive Tool for Diagnosis and Monitoring

Liquid biopsy represents one of the most transformative applications of NGS in oncology, offering a non-invasive method for tumor genotyping and disease monitoring. It primarily analyzes tumor-derived biomarkers in peripheral blood, including circulating tumor DNA (ctDNA), circulating tumor cells (CTCs), cell-free RNA (cfRNA), and extracellular vesicles (EVs) [137,138].

ctDNA, which consists of DNA fragments released by tumor cells into the bloodstream, provides a real-time molecular profile of the cancer. Its short half-life, ranging from approximately 16 min to 2.5 h, allows it to serve as a dynamic “real-time” snapshot reflecting the overall evolution of the tumor [139]. The advantages of liquid biopsy are substantial, particularly in overcoming the limitations of traditional tissue biopsies. It is non-invasive, carries a lower risk of complications compared to invasive procedures, and enables frequent sampling, which is crucial for dynamic disease monitoring [140]. Liquid biopsy, especially when combined with NGS, can overcome issues of tumor heterogeneity, as it captures genetic material from various tumor sites and facilitates the early detection of resistance mechanisms, even identifying previously unknown modes of resistance [141]. The applications of liquid biopsy are broad and transformative. Its most promising aspects lie in cancer screening and early diagnosis, which can significantly improve survival outcomes and reduce disease burden [142]. Beyond early detection, liquid biopsy is used for identifying targetable mutations, monitoring treatment response, detecting minimal residual disease (MRD) often weeks earlier than radiological imaging, and identifying disease relapse and the emergence of resistant subclones, thereby enabling real-time treatment adjustments [143,144,145]. Despite its immense promise, liquid biopsy faces several challenges. Turnaround time (phlebotomy-to-report) remains a practical bottleneck. It is influenced by sample logistics, ctDNA extraction, library prep, sequencing depth, and downstream analysis, and may delay therapy selection in rapidly progressive disease. These include the difficulty in detecting analytes in very early-stage cancers due to low tumor burden [142,146], accurately evaluating the tumor molecular fraction [42,147], and the inherent rarity of CTCs in the bloodstream. Furthermore, the purification and isolation of tumor-derived extracellular vesicles, another promising biomarker source, remain technically challenging due to their low proportion among total EVs and the need for highly sensitive and specific methods [140]. The effective implementation of liquid biopsy in large-scale population screening settings also remains a significant hurdle [142].

#### 4.1.2. Comprehensive Genomic Profiling (CGP) for Tumor Characterization

CGP represents a major innovation in the application of NGS, utilizing a single assay to simultaneously assess hundreds of genes and a broad spectrum of clinically relevant cancer biomarkers [148,149]. This approach provides unprecedented breadth and depth of sequencing, offering a holistic view of the tumor’s genetic landscape [150]. The benefits of CGP are extensive. It can detect a wide variety of genomic alterations, including SNVs, insertions and deletions (indels), CNVs, fusions/translocations, and splice variants [43]. Beyond individual mutations, CGP can also assess indicators of genomic instability, such as tumor mutational burden (TMB), microsatellite instability (MSI), and homologous recombination deficiency (HRD), all within a single assay [31,148]. This comprehensive assessment identifies more clinically relevant variants than traditional single-gene tests or more limited hotspot NGS panels [151]. CGP consolidates biomarker detection into a single multiplex assay, eliminating the need for sequential testing, which saves valuable time and precious tumor sample material [136]. Evidence from the IMPACT study and other precision oncology initiatives demonstrates that treatment outcomes significantly improve when therapy selection is guided by comprehensive molecular profiling [26]. Studies have shown that treatment outcomes can be significantly improved when therapy is initiated after genomic profiling results are available, underscoring the value of rapid CGP solutions in enabling first-line implementation of precision medicine [152]. Furthermore, major guidelines, including those from the European Society for Medical Oncology (ESMO) and the National Comprehensive Cancer Network (NCCN), now endorse CGP as a preferred strategy for patients with advanced solid tumors where molecularly matched therapies are available [43,149].

### 4.2. Guiding Personalized Cancer Treatment: Targeted Therapies and Immunotherapy

NGS is indispensable in precision oncology, enabling comprehensive identification of oncogenic drivers and resistance mechanisms to inform targeted therapy and immunotherapy selection. This molecular stratification has reshaped treatment paradigms in NSCLC, markedly improving survival outcomes for biomarker-selected patients [153]. Below, we examine how NGS-driven insights guide personalized treatment through targeted agents against oncogenic drivers and through biomarkers for immunotherapy response.

#### 4.2.1. Oncogenic Driver Mutations and Targeted Agents

The discovery of specific oncogenic driver mutations in NSCLC has revolutionized therapy, allowing the use of targeted drugs that outperform chemotherapy in appropriate patients. Key driver alterations in NSCLC and their corresponding targeted treatments include:

- *EGFR* mutations occur in ~15% of lung adenocarcinomas in Western populations and up to 50% in East Asian patients [153,154,155]. These sensitizing mutations (exon 19 deletions, *L858R*, etc.) predict a strong response to *EGFR* tyrosine kinase inhibitors (TKIs), which yield significantly higher progression-free survival compared to chemotherapy [156]. Despite initial benefit, resistance inevitably develops. A prominent resistance mechanism is the *EGFR T790M* mutation, which accounts for 50–60% of acquired resistance to first- and second-generation TKIs [157,158,159].
- Chromosomal ALK rearrangements (most commonly *EML4–ALK*) are found in ~3–5% of NSCLC cases (especially in younger non-smokers with adenocarcinoma) [160,161]. Several ALK inhibitors have transformed treatment outcomes, with alectinib emerging as a preferred first-line option after the ALEX trial demonstrated a median PFS of 34.8 months compared with 10.9 months for crizotinib [162]. Next-generation inhibitors like lorlatinib offer potent activity against CNS metastases and resistance mutations [163,164].
- *KRAS* mutations are among the most common oncogenic drivers in NSCLC, present in approximately 25–30% of lung adenocarcinomas [43,161]. The KRAS G12C mutation accounts for ~13% of all KRAS mutations in NSCLC and has emerged as a major therapeutic target. Sotorasib, evaluated in a clinical trial, demonstrated an objective response rate (ORR) of 37% and median progression-free survival (PFS) of 6.8 months in previously treated patients with *KRAS G12C*-mutated NSCLC [165]. Similarly, adagrasib, assessed in the KRYSTAL-1 study, achieved an ORR of 43% with durable responses in a similar population [166]. These data led to the accelerated approval of both sotorasib and adagrasib for advanced *KRAS G12C*-mutated NSCLC following prior systemic therapy. However, KRAS-mutated tumors often exhibit co-mutations in genes such as *STK11* or *KEAP1*, which are associated with poor response to immune checkpoint inhibitors and may require combinatorial therapeutic approaches [43].
- *BRAF* mutations occur in 1–5% of NSCLC cases, predominantly in adenocarcinoma histology [160,167]. The BRAFV600E variant is the most common, found in over 50% of BRAF-mutated cases [168]. These mutations lead to persistent activation of the mitogen-activated protein kinase (MAPK) pathway, driving uncontrolled cell growth and proliferation [169,170]. Targeted therapies, specifically BRAF/MEK inhibitors (e.g., dabrafenib in combination with trametinib), have demonstrated improved overall response rates and progression-free survival in patients with BRAFV600E-mutated NSCLC [160]. Patients with BRAFV600E mutations treated with platinum-based chemotherapy have been associated with worse outcomes [169].
- *ROS1* fusions occur in approximately 1–2% of lung adenocarcinomas and are often observed in younger patients with little to no smoking history [171]. These fusions result in constitutively activated *ROS1*, driving uncontrolled cell growth. A range of targeted therapies, including *ROS1*-TKIs like crizotinib, entrectinib, lorlatinib, taletrectinib, repotrectinib, and NVL-520, have shown significant efficacy, including activity against brain metastases and some resistant *ROS1* mutations (e.g., *G2032R*) [172,173,174]. Crizotinib was the first FDA-approved *ROS1*-targeted TKI, achieving an ORR of 72–76% and a median PFS of 15.9–19.3 months in a clinical trial [175].
- Rearrangements in the *RET* gene drive oncogenesis in 1–2% of NSCLCs and are typically found in younger, non-smoking patients [176,177]. The development of selective *RET* inhibitors, such as selpercatinib (Retevmo) [178,179] and pralsetinib (Gavreto) [180] (both FDA-approved), has dramatically improved outcomes for patients whose tumors harbor these alterations, moving beyond the modest activity of earlier multi-kinase inhibitors like cabozantinib and vandetanib [176,177].
- *MET* exon 14 skipping mutations occur in approximately 3–4% of NSCLC cases, with higher prevalence observed in older patients, women, and non-smokers, particularly in lung adenocarcinoma histology [181,182]. This alteration leads to dysregulation of the *MET* receptor tyrosine kinase and increased responsiveness to specific *MET TKIs*, including capmatinib, tepotinib, and savolitinib. Capmatinib, approved by the FDA in 2020, is a selective *MET-TKI* that has shown significant clinical activity in patients with this mutation, including some activity against CNS metastases [181].
- While less common, occurring in 2–4% of NSCLC cases, *HER2* mutations are increasingly recognized as actionable targets. Trastuzumab deruxtecan (T-DXd), an antibody-drug conjugate, has become a standard second-line therapy for *HER2*-mutated NSCLC, and novel *HER2*-targeted TKIs like zongertinib, poziotinib, and mobocertinib are emerging as promising options [183]. Comprehensive molecular testing, utilizing both tissue and liquid biopsy, is crucial to ensure these mutations are not missed [184].
- Neurotrophic tyrosine receptor kinase (*NTRK*) gene fusions are rare in lung cancer, accounting for less than 1% of cases, but are highly actionable [185,186]. TRK fusion proteins promote oncogenesis by mediating constitutive cell proliferation and survival [187]. Potent and promising TRK inhibitors, such as larotrectinib [188] and entrectinib [189], have demonstrated encouraging antitumor activity in patients with NTRK-rearranged malignancies.

#### 4.2.2. *PD-L1* and Immunotherapy Biomarkers

Beyond targeted therapies, NGS also contributes to guiding immunotherapy, particularly through the assessment of biomarkers like programmed death-ligand 1 (*PD-L1*) expression [190]. *PD-L1* tumor expression is a validated target for immune checkpoint inhibition in advanced malignancies. When expressed on cancer cells, it interacts with its receptor PD-1 on T cells, thereby suppressing T cell activation and allowing the tumor to evade the anti-tumor immune response [191]. This interaction leads to T-cell exhaustion and tolerance in the tumor microenvironment, enabling prolonged tumor progression and survival.

Higher *PD-L1* expression is associated with improved outcomes in NSCLC treated with checkpoint inhibitors, as demonstrated in several pivotal trials, including KEYNOTE-024 [192], KEYNOTE-042 [193], IMpower110 [12], and OAK [194]. In the KEYNOTE-024 trial, pembrolizumab monotherapy significantly improved progression-free and overall survival compared with chemotherapy in treatment-naïve advanced NSCLC patients with *PD-L1* tumor proportion score (TPS) ≥50% and no EGFR/ALK alterations [192]. This established a ≥50% TPS cut-off as the benchmark for first-line monotherapy. KEYNOTE-042 extended these findings, demonstrating that pembrolizumab provided a survival benefit over chemotherapy in a broader cohort of patients with PD-L1 TPS ≥1%, though the greatest benefit was observed in the ≥50% subgroup [193]. Similarly, the IMpower110 trial confirmed that atezolizumab monotherapy significantly improved overall survival compared with chemotherapy in patients with high PD-L1 expression (TC3 or IC3), validating *PD-L1* as a predictive biomarker across different checkpoint inhibitors [12]. In the second-line setting, the CheckMate 057 trial showed that nivolumab significantly improved overall survival over docetaxel in previously treated nonsquamous NSCLC, with greater benefit in patients with higher *PD-L1* expression [195].

However, the utility of *PD-L1* as a predictive biomarker is nuanced and faces several challenges. The relationship between *PD-L1* expression and therapeutic benefit to anti-PD-1/PD-L1 therapy is often imprecise. Some patients with low or no *PD-L1* expression can still derive durable benefit from immunotherapy, indicating that *PD-L1* expression alone may be insufficient for definitive patient selection [196]. This suggests that a combination of factors, rather than *PD-L1* expression in isolation, may be necessary to accurately inform treatment decisions and predict response to anti-PD-1/PD-L1 therapies.

Challenges also exist in the variability of *PD-L1* assessment methods, including different assays (e.g., Dako 28-8 assay) and cut-off points (e.g., 1%, 5%, 10%, 50%), which can lead to imprecise relationships between expression levels and therapeutic benefit [191,193]. Furthermore, PD-L1 expression can exhibit significant intra-tumor heterogeneity (within the same tumor) and inter-tumor heterogeneity (between primary and metastatic sites or different paraffin blocks from the same resected specimen), leading to sampling errors and potential misclassification of *PD-L1* status [197]. Discordance rates can be as high as 31.4% between biopsy and resected specimens, and 35.8% between different paraffin blocks [198]. The dynamic nature of *PD-L1* expression, which can be induced by factors like interferon-gamma (IFNγ), also adds to the complexity of its assessment [198,199].

Given these limitations, researchers are actively investigating additional biomarkers of immunotherapy response, often using NGS to derive comprehensive genomic and immune profiles:

- Tumor mutational burden (TMB) measures the total number of nonsynonymous mutations in a tumor genome [43,148,196,200]. High TMB can indicate a greater likelihood of neoantigen formation, potentially leading to a stronger immune response. In some cases, a high TMB might be a consideration for immunotherapy even if PD-L1 expression is negative [196,200].
- Microsatellite instability (MSI) high status, caused by defects in DNA mismatch repair, is a strong predictor of immunotherapy benefit in tumors like colorectal cancer. MSI-high is rare in NSCLC, but comprehensive genomic testing can detect it when present [43,148]. Like TMB, MSI is a marker of genomic instability and high neoantigen load. Pembrolizumab has tissue-agnostic approval for MSI-high tumors [190], though MSI-high NSCLC is extremely uncommon [201].
- Specific genetic alterations can influence tumor immune resistance. For example, KRAS-mutated tumors co-mutated with *STK11* or *KEAP1* are often highly immune resistant to single PD-1 checkpoint inhibition, suggesting that combination immunotherapy might be more beneficial in such cases [202,203].
- The presence and characteristics of immune cell infiltrates within the tumor microenvironment are also being investigated as potential biomarkers [199].
- Liquid biopsy techniques, enabled by NGS, are being explored for the discovery of novel circulating biomarkers that can predict immunotherapy response [144,147].

Artificial intelligence (AI) tools are increasingly used to advance immunotherapy biomarker assessment. Automated PD-L1 quantification on whole-slide images has been shown to significantly reduce interobserver variability compared with manual scoring [133,197,204]. Future research aims to leverage AI to directly analyze histology slides to predict PD-L1 status or even forecast immunotherapy response. NGS has also expanded the number of actionable oncogenic driver mutations beyond initial discoveries such as EGFR, ALK, and KRAS, enabling the development of a growing arsenal of highly specific targeted therapies for different lung cancer subtypes [153,190,205]. However, the predictive value of biomarkers like PD-L1 remains complex and context-dependent, underscoring the need for integrated, multi-biomarker approaches to improve patient stratification and therapeutic decision-making. Table 4 summarizes predictive immunotherapy biomarkers—PD-L1, TMB, MSI, genomic alterations, immune infiltrates, and circulating biomarkers—along with assessment methods, implications, and limitations.

### 4.3. Monitoring Drug Resistance and Disease Progression

Drug resistance represents a persistent and significant challenge in the management of advanced NSCLC patients, often serving as a primary cause of treatment failure [206].

#### 4.3.1. Mechanisms of Drug Resistance

Drug resistance can manifest through various mechanisms, broadly categorized into primary (intrinsic) and secondary (acquired) resistance. Primary resistance refers to the inherent presence of tumor characteristics or gene alterations that confer insensitivity to specific therapeutic agents even before treatment initiation [207]. This can be due to several factors, including increased drug efflux mediated by the upregulation of ATP-binding cassette (ABC) transporters like ABCB1, ABCC1, and ABCG2, which actively pump drugs out of cancer cells, thereby reducing intracellular drug concentrations [208]. Cancer stem cells (CSCs) often contribute to this intrinsic resistance by exhibiting high expression of these transporters [208,209]. Other mechanisms include decreased drug uptake, such as the downregulation of copper transporters like CTR1/2 for platinum agents [210], and increased drug metabolism or inactivation through enhanced activity of enzymes like glutathione S-transferase (GST) and increased synthesis of glutathione, which facilitates detoxification and efflux of drugs [211].

Furthermore, primary resistance can arise from inherent resistance to DNA damage, which is the primary target for many chemotherapeutic agents like cisplatin. Cancer cells may upregulate sophisticated DNA repair mechanisms to negate the cytotoxic effects of these drugs [212,213]. The nucleotide excision–repair (NER) pathway, involving key enzymes like excision-repair cross-complementary group 1 (ERCC1), removes drug-induced DNA adducts (e.g., platinum-DNA adducts), allowing DNA polymerase to repair the damage [212,214]. Low ERCC1 expression has been correlated with prolonged survival in NSCLC patients treated with cisplatin plus gemcitabine [212,215]. The mismatch-repair (MMR) pathway, while typically maintaining genomic stability, can also repair DNA–platinum adducts, and defects in this pathway are associated with resistance to methylating agents [211,214,216]. The base excision–repair (BER) pathway, initiated by DNA glycosylases, recognizes and catalyzes the removal of damaged DNA bases, and its inhibition can increase sensitivity to alkylating agents [217,218]. Additionally, evasion of apoptosis through alterations in pro-apoptotic and anti-apoptotic B-cell lymphoma-2 (Bcl-2) proteins can inhibit cell death pathways, allowing resistant cells to survive. Epithelial–mesenchymal transition (EMT) also contributes to primary resistance by leading to a cancer stem cell-like phenotype associated with increased invasiveness, stemness, and chemoresistance [219].

In contrast, secondary resistance, also known as acquired resistance, develops during therapy after an initial response. This typically occurs due to the emergence of new mutations or amplification of resistance genes, leading to a gradual decrease in the effectiveness of initially useful agents, or rendering them completely ineffective [159]. A prominent molecular example of acquired resistance is the *EGFR* T790M mutation, which accounts for 50–60% of cases of resistance to first- and second-generation *EGFR* TKIs [159]. Lazertinib, a third-generation, covalent and mutant-selective *EGFR* TKI with good CNS penetration, targets activating and T790M mutations while largely sparing wild-type *EGFR*. It was first approved (Korea, Jan 2021) at 240 mg once daily for *EGFR* T790M-positive NSCLC after prior EGFR-TKIs. In the phase 1/2 LASER201 study, lazertinib achieved an ORR ~55% and median PFS ~11.1 months with intracranial activity in pretreated T790M-positive disease [220]. This gatekeeper mutation increases the affinity for ATP in the ATP-binding domain of EGFR, hindering the binding of ATP-competitive kinase inhibitors [159]. While first-generation TKIs are affected, third-generation TKIs like Osimertinib can overcome this resistance by irreversibly binding to a different cysteine residue in the ATP pocket [159]. Loss of the T790M mutation is often associated with earlier resistance and poorer survival, frequently linked to the development of KRAS mutations, gene fusions, or histological transformation [159,202]. Another example is *MET* amplification, which can also contribute to resistance to EGFR inhibitors [221]. Dual-pathway inhibition strategies, such as combining *EGFR* TKIs with *MET* inhibitors (Amivantamab, a bispecific antibody targeting *EGFR* and *MET*, combined with Lazertinib), are being explored to address these resistance mechanisms before they emerge [222]. Furthermore, drug-resistant cancers often show markedly altered DNA methylation and histone acetylation patterns, such as hypermethylation of tumor suppressor genes like *RASSF1A* and *CDKN2A* [211], and hypoacetylation of histones H3 and H4, which can promote drug resistance by silencing important genes [223]. Changes in the tumor microenvironment (TME), including elevated expression of extracellular matrix (ECM) components, high numbers of cancer-associated fibroblasts (CAFs), and increased concentrations of immunosuppressive cells like myeloid-derived suppressor cells (MDSCs), also play a significant role in acquired drug resistance by inhibiting anti-tumor immunity [199].

Overall, drug resistance in NSCLC is a complex process influenced by a dynamic interplay of genetic, epigenetic, and microenvironmental factors.

#### 4.3.2. NGS and Liquid Biopsy for Real-Time Resistance Detection

NGS, when combined with liquid biopsy, offers a powerful and non-invasive method for monitoring the dynamic evolution of drug resistance in NSCLC. Serial sampling via ctDNA enables frequent molecular surveillance, facilitating early detection of emerging resistance mechanisms—even those not apparent through standard imaging or tumor biopsies [145].

Analysis of ctDNA through NGS can detect residual disease weeks earlier than conventional radiological imaging. This early detection is critical, as ctDNA-positive patients are at a higher risk of relapse and often exhibit worse outcomes [137,145]. Furthermore, ctDNA analysis facilitates the dynamic monitoring of clonal evolution within the tumor and helps identify the emergence of resistant subclones, enabling clinicians to make timely treatment adjustments and adapt therapeutic strategies as new resistance mechanisms emerge [137,139]. This continuous, non-invasive genetic surveillance is essential for adapting therapies to the dynamically evolving nature of cancer, moving beyond a static view of the disease.

The successful use of next-generation sequencing in hematological cancers provides an important reference point for lung cancer care. In leukemias and lymphomas, NGS is now considered the gold standard for minimal residual disease (MRD) monitoring, with greater sensitivity than traditional methods such as flow cytometry or RT-qPCR [224,225]. By detecting very small populations of residual malignant cells, NGS makes it possible to anticipate relapse and intervene earlier [226,227]. Applying similar principles to lung cancer could strengthen current approaches to surveillance and treatment adaptation.

A further advantage of NGS is its broad ability to detect multiple mutations simultaneously. Targeted sequencing panels can capture a wide spectrum of clinically relevant changes, providing a more complete picture of the tumor genome compared with conventional single-assay methods [228,229]. This is highly relevant in NSCLC, where drug resistance may arise through diverse pathways involving genes such as *EGFR* or *ALK*. Incorporating NGS into clinical practice, therefore, enhances the ability to identify resistance early and supports more precise therapeutic decision-making [230].

Beyond its role in tracking resistance mechanisms, NGS applied to plasma cell-free DNA (cfDNA) offers additional clinical advantages. Because cfDNA sequencing is non-invasive, it provides a real-time snapshot of tumor genetics and can detect actionable mutations in key driver genes such as *EGFR*, *ALK*, and *MET* [231]. Identifying these alterations early is particularly valuable for selecting or adjusting targeted therapies, and several studies have shown that cfDNA-based NGS can reveal resistance mutations that warrant a timely change in treatment [232]. In the postoperative setting, cfDNA analysis is also useful for detecting persistent or newly emerging mutations, giving oncologists critical information for tailoring treatment to the tumor’s evolving molecular profile [233].

Liquid biopsy approaches add another dimension to this strategy. Circulating tumor DNA (ctDNA) analysis through NGS has already proven its value in hematology and is gaining traction in solid tumors. In lung cancer, ctDNA profiling enables non-invasive monitoring of treatment response and tumor evolution [222,234]. Although not yet routine in clinical care, growing evidence suggests that ctDNA can predict relapse and resistance with high sensitivity [235,236]. Taken together, these developments point toward a future in which lung cancer management relies on continuous molecular surveillance, allowing therapy to be adapted in real time as resistance emerges.

## 5. Challenges and Limitations in Clinical Implementation of NGS

Despite its transformative potential, the widespread clinical implementation of NGS faces several significant challenges and limitations, encompassing technical hurdles, economic complexities, data interpretation difficulties, and issues related to standardization and workflow integration [43,45,119,134].

### 5.1. Technical and Methodological Hurdles

The technical and methodological aspects of NGS present inherent challenges that can impact the quality and reliability of genomic data in a clinical setting.

A primary concern is sample quality. Degraded or contaminated samples, particularly common with FFPE tissues, can lead to suboptimal library quality and subsequent sequencing failures [121,237]. RNA, being inherently less stable, is especially prone to degradation, necessitating meticulous handling and preservation techniques [125]. To mitigate these issues, careful optimization of fragmentation time and increased DNA input for low-quality samples are often required [45,128].

Coverage uniformity across the genome remains a technical hurdle. While WGS aims for comprehensive coverage, short-read sequencing technologies, which are predominantly used in most clinical NGS applications, have inherent limitations [81]. These limitations manifest in difficulty analyzing complex structural variants (e.g., those larger than 300–400 base pairs), long repeat sequences [40], triplet repeat expansions [52], polyploidy [36,52], mosaicism [51,87,121], and variants located in regions with high sequence identity (such as paralogous genes and pseudogenes) or high guanine-cytosine (GC) content [81,121]. This means that despite the high throughput, there are intrinsic technical boundaries that prevent a perfectly uniform genomic readout, especially for highly complex or rare alterations. The gap between what NGS technology is theoretically capable of detecting and what it can reliably and consistently identify for clinical decision-making is a significant hurdle that requires ongoing technological refinement and validation [125]. These technical constraints directly impact the comprehensiveness and accuracy of genomic profiling, necessitating continuous optimization and the development of complementary technologies to achieve a more complete molecular picture.

### 5.2. Cost-Effectiveness and Reimbursement Complexities

Despite the dramatic reduction in the cost per base of sequencing over the past decade (a five-fold decrease in 10 years, with whole-genome sequencing now commercially available for less than $1000 [80]), the overall cost of comprehensive NGS testing remains a substantial barrier to its widespread clinical adoption [134,238,239]. The high costs are not solely attributed to the sequencing reaction itself but also encompass reagents, specialized sequencing machines, and critically, the extensive resources required for data storage, transfer, processing, and sophisticated bioinformatics analysis [77]. For instance, targeted gene panels can range from €376 to €968 in France [238] and average $1609 per sample in the USA [239].

Beyond the direct costs, reimbursement processes for NGS testing are notably complex and often inconsistent [43,78,134,238,239]. Insurance coverage policies for multi-gene testing frequently lag behind evolving clinical guidelines [43,134], creating a significant disconnect between recommended clinical practice and financial accessibility [135]. Payer frameworks, originally designed for single-gene assays, often lack the flexibility to adequately account for the integrative benefits of NGS, leading to uncertainties around clinical utility from a payer perspective [43]. This economic and regulatory disconnect means that even when NGS offers clear clinical value, its widespread and equitable adoption is hindered by financial and policy challenges, limiting patient access to these advanced diagnostic tools [134]. This underscores the need for updated insurance coverage policies that align with both clinical and economic evidence, as studies suggest NGS testing can be cost-saving and cost-effective in the long run [78,79,239].

### 5.3. Data Interpretation, Bioinformatics Expertise, and Incidental Findings

The sheer volume and complexity of data generated by NGS pose significant challenges for accurate and consistent interpretation. Without robust bioinformatics pipelines, the vast data output from NGS would be essentially unusable [119,121,130]. Interpreting NGS results requires a high level of expertise, combining sophisticated bioinformatics tools with nuanced clinical judgment [43,131,134].

A major concern is the generation of many variants of uncertain significance. These are genetic alterations for which there is currently insufficient evidence to definitively classify them as pathogenic or benign [118,119,128,130]. The presence of numerous VUS can cause considerable confusion among clinical geneticists and clinicians, complicating treatment decisions and patient counseling.

Furthermore, NGS increases the likelihood of incidental findings, which are unexpected genetic alterations discovered during sequencing that are unrelated to the primary clinical question. Managing and communicating these findings ethically and effectively presents complex challenges related to informed consent, patient privacy, and the appropriate return of results [89]. The distinction between diagnostic and screening tests can become blurred, raising questions about how clinicians should best inform patients of such unexpected information.

The current landscape also reveals a significant expertise gap. There is a notable lack of adequate staff training and knowledge among clinicians regarding NGS testing [43,45,134]. This often translates to clinicians being less confident in ordering or interpreting NGS tests and subsequently less likely to recommend treatments or clinical trial opportunities based on the results [43,134,135]. This human capacity to accurately interpret, contextualize, and communicate complex genomic findings remains a critical bottleneck in scaling NGS adoption and ensuring consistent, high-quality patient care [131,134]. Technology’s rapid advancement has outpaced the development of the necessary human expertise and ethical frameworks required to fully leverage its capabilities [43,134].

### 5.4. Standardization and Clinical Workflow Integration

The successful integration of NGS into routine clinical practice is further hampered by a lack of standardization across laboratories and the complexities of clinical workflow integration [43,119]. Currently, there is an absence of standardized procedures within laboratories for NGS, which can lead to significant variations in results between experiments [85,119]. This variability limits the reproducibility of findings and hinders the comparability of results across different research studies and clinical centers [85,136]. Variations can occur at multiple stages, including library preparation, the choice of sequencing platforms, and the specific data analysis pipelines employed [45,119,134].

The rapid transition of NGS from a research tool to a clinical diagnostic application has often occurred with the development of laboratory-developed techniques [43,45,119]. This rapid transfer, while beneficial for innovation, has made it difficult to accurately assess the gain in production relative to the true cost, contributing to the economic complexities previously discussed [238,239].

Integrating NGS effectively into routine clinical workflows requires addressing critical issues such as turnaround times and streamlining complex processes [43,45]. For advanced-stage cancer patients, rapid results are paramount for timely treatment decisions [43]. Achieving faster NGS solutions while maintaining data quality and reproducibility is a continuous challenge for clinical research laboratories [85,119,136]. The need for streamlined, automated workflows with minimal manual intervention is clear to enhance reproducibility, minimize errors, and produce consistent, high-quality libraries more efficiently [85]. The transition of NGS from a research-driven, variable application to a standardized, reliable, and integrated clinical diagnostic tool requires substantial effort in harmonizing protocols, implementing robust quality control measures, and developing standardized data interpretation guidelines across institutions [118,130].

## 6. Future Directions and Emerging Technologies in Cancer Genomics

Cancer genomics is advancing rapidly, with new platforms poised to complement, and in some contexts surpass, conventional short-read NGS. Long-read and duplex error-corrected sequencing [51,52], single-cell and spatial multi-omics [240,241,242,243], and methylation/chromatin-accessibility assays address gaps in repeat expansions, complex structural variants, phasing, and epigenetic regulation [63,64], while tumor-informed liquid biopsy for minimal residual disease [137,138,139,140] and AI-enabled analytics [131,132,134] accelerate translation of data into clinically actionable insights.

### 6.1. AI and Machine Learning (ML) in NGS Data Analysis and Treatment Prediction

AI and ML, a subfield of AI, are rapidly becoming foundational components in modern oncology, offering sophisticated tools across the entire spectrum of cancer care. These technologies are particularly impactful in their ability to analyze vast volumes of data more efficiently and enhance diagnostic accuracy [131,132].

ML models can be trained on large datasets of previously curated genomic information to predict whether specific patient variants should be reported in clinical oncology reports [130,131]. This capability provides invaluable decision support for expert curators, significantly reducing inter-reviewer variability and ensuring more consistent reporting practices [118,130]. By automating variant classification and reducing manual interpretation time, AI/ML can help overcome the bottleneck associated with expert genomic analysis, thereby scaling the volume of patients who can benefit from comprehensive genomic assessment [131,132].

On a broader level, tools like CUP-AI-Dx and Pan-Cancer ML Classifiers extend beyond single tumor types, applying RNA-seq to solid cancers more generally. By integrating machine learning, these methods process large-scale gene expression data to uncover molecular signatures shared across multiple cancers. Such frameworks facilitate the discovery of biomarkers with prognostic value [244,245]. More recent developments incorporate artificial intelligence to improve classification accuracy and diagnostic performance—an important step given the complexity of cancer genomics [246].

AI tools demonstrate considerable performance in recognition, linkage, and outcome prediction based on medical images, which are integral to multidisciplinary team functions in oncology [133,197,204]. For instance, AI has successfully validated automated PD-L1 reading, improving interobserver concordance among pathologists [133,190,191]. The future holds potential for AI to directly decipher undiscovered morphology from standard histology slides, predicting PD-L1 status or even directly forecasting immunotherapy response [133,204]. Furthermore, AI facilitates drug discovery by identifying potential therapeutic targets and accelerating the development of new agents, supports individualized treatment planning, and enhances clinical decision support systems, thereby transforming precision cancer therapies [132,134]. AI and ML are thus poised to significantly enhance the utility of NGS by addressing key challenges in data analysis and interpretation, accelerating the translation of complex genomic data into actionable clinical insights [134,135].

### 6.2. Advanced Genomic Profiling: Single-Cell NGS and Spatial Transcriptomics

While traditional NGS provides an average genomic profile from a bulk tumor sample, emerging advanced genomic profiling techniques are moving towards a more granular understanding of cancer by dissecting its intricate micro-complexity [134,135].

#### 6.2.1. Single-Cell RNA Sequencing (scRNA-Seq)

ScRNA-seq is rapidly emerging as a powerful method for dissecting human tumor tissue at single-cell resolution, providing profound insights into carcinoma biology [240]. This technology allows for the characterization of the epigenome, transcriptome, and genome of individual cells, which is crucial for understanding the changes that occur at various stages of cancer [247,248].

ScRNA-seq has transformed the study of tumor biology by making it possible to examine gene expression at the resolution of individual cells. This approach has provided critical insights into cellular heterogeneity, lineage relationships, and intercellular communication. Recent technological advances—through platforms such as 10x× Genomics, Illumina, BD, and MGI—have enabled high-throughput applications and improved resolution, expanding the scope of scRNA-seq studies. Nonetheless, each platform carries inherent trade-offs that must be carefully considered.

By capturing transcriptional profiles at single-cell resolution, scRNA-seq reveals the full extent of cellular diversity within tumors and the complex interactions that shape tumor behavior and patient outcomes. For example, Wang et al. applied scRNA-seq to non-small cell lung cancer and identified distinct metabolic states and developmental pathways within T-cell populations—differences that critically influence antitumor immune responses [249]. Such fine-grained profiling is vital for identifying biomarkers of immune modulation and for pinpointing new therapeutic targets in immuno-oncology.

Building on these insights, scRNA-seq has also emerged as a powerful tool for dissecting the tumor microenvironment, overcoming the limitations of bulk RNA-seq. By resolving cellular heterogeneity and mapping immune landscapes, it clarifies the functional roles of individual populations in shaping tumor progression. This resolution is particularly valuable for evaluating immunotherapy outcomes and uncovering mechanisms of resistance [250,251]. Importantly, pan-cancer studies demonstrate that scRNA-seq can uncover patterns missed by bulk approaches, ultimately supporting the development of more precise treatment strategies.

Spatial transcriptomics adds another important layer by retaining information about how cells are arranged within the tumor tissue. This approach allows researchers to visualize where specific cell types are located and how they interact with each other. For instance, Song et al. combined spatial transcriptomics with imaging to provide a clearer picture of cell positioning and communication within the tumor microenvironment [252]. Similarly, work by Peng et al. demonstrated how cancer-associated fibroblasts interact with immune cells in ways that influence tumor progression and treatment response [253].

Bringing scRNA-seq and spatial transcriptomics together creates a more complete view: scRNA-seq defines the transcriptomic identity of individual cells, while spatial transcriptomics shows how those cells are organized and connected within tissues. When combined, these methods reveal how specific cellular interactions affect immune activity and therapy outcomes. As Kinker et al. emphasized, unraveling these dynamics is key to developing more effective immunotherapies and to targeting the tumor microenvironment more precisely in cancer treatment [248].

A major challenge in scRNA-seq is the high degree of technical noise in the data. The stochastic nature of transcript capture often results in dropout events, where certain transcripts—particularly those expressed at low levels—go undetected. These issues are further complicated by variability introduced during library preparation, which can undermine reproducibility and reduce the accuracy of expression quantification [254]. In addition, cell processing steps involving fragmentation and amplification may distort transcript representation, introducing biases that affect downstream biological interpretation [254].

Comparisons across scRNA-seq platforms have shown notable performance differences. For instance, Illumina sequencing supports high-throughput, cost-effective analysis of large sample sets but is largely restricted to short-read data. In contrast, long-read technologies from Pacific Biosciences (PacBio) and Oxford Nanopore can resolve complex isoforms and capture full-length transcripts. However, these methods come with lower throughput and higher costs [255]. Efforts by platforms such as 10x Genomics aim to balance these needs by providing scalable, high-throughput transcriptomic profiling with potential compatibility for long-read applications, though further evaluation is needed to substantiate such claims [256].

Recent progress in tumor genetic profiling through RNA-seq has driven the creation of a wide range of analytical tools that deepen our understanding of tumor heterogeneity and treatment responses across cancer types. In acute lymphoblastic leukemia (ALL), tools such as ALLsort and clinALL illustrate how RNA-seq can sharpen our view of the genomic landscape in hematologic cancers. These platforms have been especially valuable in pinpointing genetic alterations and fusion transcripts that define distinct ALL subtypes, which are essential for guiding personalized treatment strategies [257,258].

A further innovation has been the use of multi-omics approaches, which combine RNA-seq with complementary data types such as ATAC-seq and DNA methylation. This strategy provides a more complete picture of tumor biology by uncovering transcriptional regulatory networks involved in cancer development and progression. These integrative methods are proving crucial in identifying mechanisms behind treatment resistance and tumor evolution [259,260]. For instance, combining chromatin accessibility with transcriptomic data has revealed regulatory interactions that play a role in therapeutic response across cancers [261,262].

The field is also moving toward integrative single-cell approaches that combine transcriptomics with other single-cell technologies to better map tumor microenvironments. Methods like Microwell-seq and barcoding strategies expand the ability to capture diverse cellular populations. At the same time, they generate highly complex datasets that present new challenges in interpretation [263]. Emerging technologies are beginning to reduce costs and improve data quality, but the lack of standardization across platforms remains a barrier to reproducibility and cross-study comparisons [264,265].

Another promising direction involves targeted RNA sequencing with synthetic long reads, which has been applied to detect isoform-level alterations in cancer. This highlights the versatility of scRNA-seq in distinguishing transcript variants relevant to tumor progression. Still, such methods can be labor-intensive and often require complementary short-read sequencing to correct for error rates that remain common in long-read platforms [74].

By analyzing individual cells, scRNA-seq offers unprecedented insights into tumor heterogeneity, the dynamics of tumor stem cells, mechanisms of tumor drug resistance, and the complex interactions within the tumor microenvironment [241,266]. This fine-grained approach enables in-depth studies of intra-tumor genetic heterogeneity and facilitates computational inferences of subclonality, which are critical for understanding why treatments fail and for developing more effective, targeted therapeutic strategies [248,267].

#### 6.2.2. Spatial Transcriptomics

Spatial transcriptomics is a cutting-edge technology that enables the analysis of gene expression within tissue samples while meticulously preserving their spatial context [241,242,268]. This innovative approach opens new avenues for understanding the complex interactions that occur within the tumor microenvironment, which is known to play a crucial role in cancer progression and response to therapy [241].

By capturing the spatial coordinates alongside gene expression data, spatial transcriptomics provides insights into tumor heterogeneity at a contextualized level, revealing how gene expression varies across different regions of a tumor [269]. It also allows researchers to analyze cell–cell interactions and communication within the TME by examining co-expression patterns of genes involved in signaling pathways [243]. This technology holds immense promise for deepening the understanding of the tumor microenvironment and identifying novel therapeutic targets that are spatially dependent, thereby extending the reach of cancer immunotherapies to new patient populations [270].

### 6.3. Multi-Omics Approaches for Holistic Cancer Understanding

The understanding of cancer is moving beyond a gene-centric view to a more holistic perspective through multi-omics approaches [271,272]. These novel frameworks integrate multiple “omics” datasets—including genomics, transcriptomics, proteomics, metabolomics, and epigenomics—generated from the same patients [273]. This comprehensive integration provides a significantly more complete understanding of the intricate molecular mechanisms driving cancer [271,272].

Multi-omics studies aim to overcome the complexities arising from the genetic and phenotypic heterogeneity of cancer. By analyzing data across different molecular layers, these approaches can lead to better identification of clinical subtypes, improved prediction of survival outcomes, and a deeper understanding of key pathophysiological processes [273,274,275]. They are instrumental in predicting effective combination therapies and identifying novel predictive biomarkers, ultimately increasing response rates to targeted treatments [275]. This comprehensive view is essential for dissecting the complex molecular mechanisms driving cancer, identifying novel biomarkers, predicting therapeutic responses, and ultimately designing more effective, multi-faceted personalized treatment strategies that account for the full spectrum of cellular alterations [271,273].

### 6.4. Integration of Nanotechnology in Cancer Diagnostics and Therapeutics

Nanotechnology, defined as the control and manipulation of materials at the atomic or molecular level (typically 1–100 nm), offers groundbreaking solutions to various biomedical challenges, including those in cancer diagnosis and treatment [276,277]. This field promises to deliver quick, safe, cost-effective, and efficient methods for comprehensive cancer management [277].

In diagnostics, nanoparticles are being developed to capture and detect cancer biomarkers such as exosomes, circulating tumor cells, ctDNA, and cancer-associated proteins with high precision [278]. Nanotechnology-based diagnostic tools, including quantum dots and nanosensors, enable highly sensitive detection of cancer biomarkers and facilitate in vivo molecular imaging, offering real-time and non-invasive tumor visualization [279].

In therapeutics, nanocarriers such as liposomes, dendrimers, and polymeric nanomaterials are being engineered to enable targeted drug delivery. These nanocarriers can encapsulate chemotherapeutic agents, genes, proteins, or small molecules and deliver them directly to tumor sites, thereby improving therapeutic efficacy while significantly reducing systemic toxicity and minimizing damage to healthy tissues [277,278]. Furthermore, nanotechnology facilitates the concept of “theragnostic,” where multifunctional nanoparticles combine both therapeutic and diagnostic capabilities within a single agent, allowing for simultaneous diagnosis and treatment [280]. This integration promises to bridge the gap between molecular understanding (from NGS) and effective physical intervention, leading to more effective, less toxic, and highly personalized diagnostic and therapeutic interventions.

## 7. Conclusions

NGS has firmly established itself as one of the most transformative technologies in oncology, reshaping how cancers are diagnosed, monitored, and treated. Its ability to comprehensively profile tumors at the molecular level has allowed clinicians to uncover key genetic drivers, personalize treatment strategies, and improve patient outcomes in ways that were unimaginable just a decade ago. Beyond identifying actionable mutations, NGS is playing an increasing role in detecting hereditary cancer syndromes, monitoring minimal residual disease, and guiding immunotherapy through the discovery of predictive biomarkers. The growing use of liquid biopsy has further enhanced its utility, providing a minimally invasive and dynamic approach to capturing tumor evolution and emerging resistance mechanisms in real time.

Yet, the full integration of NGS into routine cancer care remains a work in progress. Technical challenges such as variable sample quality, uneven genomic coverage, and limitations in detecting certain variant types continue to constrain its clinical scope. Economic barriers, including high costs and fragmented reimbursement policies, hinder equitable access. The interpretation of complex genomic data, complicated by variants of uncertain significance and incidental findings, requires significant bioinformatics expertise and standardized protocols, which are still under development in many centers.

Looking ahead, the field is rapidly advancing. Artificial intelligence and machine learning are poised to streamline data analysis and variant interpretation, while single-cell sequencing and spatial transcriptomics promise deeper insights into tumor heterogeneity and the tumor microenvironment. Multi-omics approaches will integrate diverse molecular layers to refine patient stratification, and nanotechnology offers innovative possibilities for targeted drug delivery and theranostics. Together, these innovations bring us closer to a future where molecularly guided, patient-centered oncology becomes the standard of care, ensuring that the right treatment reaches the right patient at the right time.

## Figures and Tables

**Table 1:** Comparative features of Sanger sequencing and NGS for clinical and research applications.

Aspect	Sanger Sequencing [46]	Next-Generation Sequencing (NGS) [35,36,52]
Throughput	Single DNA fragment at a time	Massively parallel; millions of fragments simultaneously
Sensitivity (detection limit)	Low (~15–20%)	High (down to 1% for low-frequency variants)
Cost-effectiveness	Cost-effective for 1–20 targets, high for large regions	Cost-effective for high sample volumes/many targets
Discovery power	Limited; interrogates a gene of interest	High; detects novel or rare variants with deep sequencing
Read length	Typically, up to 1000 base pairs	Short (75–300 bp) to Ultra-long (100,000+ bp)
Workflow	Labor-intensive, serial processing	High-throughput, automated workflows
Data output	Small, limited DNA snapshot	Massive datasets, comprehensive genomic coverage
Primary use	Validation of NGS results, single gene analysis	Comprehensive genomic profiling, discovery, and large-scale studies
Turnaround time	Years for the whole genome	About a week for the whole genome
Variant detection capability	Limited to specific regions; single gene analysis	Single-base resolution; detects SNPs, indels, CNVs, SVs, and large chromosomal rearrangements

**Table 2:** Technical and practical differences between Illumina, ONT, and PacBio sequencing platforms.

Aspect	Illumina [35,36,46]	Oxford Nanopore Technologies (ONT) [52,59,60]	Pacific Biosciences (PacBio) [61,64,66]
Sequencing chemistry/Principle	Sequencing by Synthesis (SBS)	Nanopore sequencing (direct reading of single molecules)	Single-Molecule Real-Time (SMRT) sequencing
Read length	Short (typically 75–300 bp)	Ultra-long (tens of thousands to 100,000+ bp)	Long (up to 100,000 bp, routinely 15–25 kb HiFi reads)
Accuracy/Error rate	High (0.1–0.5%)	Traditionally higher (10–15%), improving with Q20+ chemistry and duplex reads	Traditionally higher (10–15%), improving with HiFi reads (circular consensus sequencing)
Throughput	Very high (millions of reads per run)	Variable, real-time data acquisition	Relatively lower than Illumina
Cost-effectiveness	Most cost-effective for short-read sequencing (per Gb often < $50)	Improving with newer platforms (e.g., PromethION)	Improving with newer platforms (e.g., Revio)
Key applications	Genome resequencing, transcriptomics, genome-wide association studies (GWAS), WES, RNA-seq, ChIP-seq, Hi-C	De novo genome assembly, structural variant detection, haplotype phasing, rapid diagnostics, telomere-to-telomere sequencing, repeat-rich regions	Full-length transcript sequencing, metagenomics, epigenomics, large indel detection, complex genomic regions (high GC, repetitive)
Bioinformatics complexity	Easier to work with, mature, and stable tools	More challenging initially, but improving tools, pipeline design depends on the project goal	More challenging initially, but improving tools, pipeline design depends on the project goal

**Table 3:** Key features of major NGS approaches (WGS, WES, RNA-Seq) in clinical and research settings.

NGS Type	Targeted Region	Primary Applications	Key Advantages	Key Limitations
WGS [78,81,82]	Entire human genome	Comprehensive variant detection (SNPs, indels, SVs, CNVs), identification of disease-influencing factors, and disease evolution tracking	Most comprehensive genomic coverage, unbiased detection of variants across coding and non-coding regions	Highest cost, massive data volume requiring extensive storage and analysis, challenges in interpreting non-coding variants, limitations in detecting certain complex structural variants or repeats
WES [83,87,88]	Protein-coding exons (exome) (~1% of genome)	Identifying genetic causes of rare diseases, detection of known disease-causing variants in coding regions	More cost-effective than WGS, focused on clinically relevant coding regions, generates fewer variants of uncertain significance (VUS) than WGS	Misses variants in non-coding regions (except canonical splice sites) [25], less reliable for detecting Copy Number Variants (CNVs) and large structural rearrangements, may not detect mosaicism reliably, technical challenges in some regions (pseudogenes, repeats), risk of incidental findings
RNA-Seq [90,93,96,99]	RNA transcripts (gene expression)	Gene expression profiling, detection of alternative splicing and transcript isoforms, fusion gene detection, biomarker discovery	Provides dynamic view of gene activity, detects novel transcripts, provides insights into regulatory elements and pathways	RNA prone to degradation, library preparation biases, data complexity, and interpretation challenges, may not fully represent post-transcriptional modifications, lack of standardization affects reproducibility, costly, onerous, and time-intensive workflow

**Table 4:** Overview of predictive biomarkers for immunotherapy.

Biomarker	Role/ Mechanism	Assessment Method	Clinical Implications	Challenges/ Limitations
*PD-L1* expression [12,191,192,193,194]	Ligand binding to PD-1 on T cells, suppressing immune response and promoting immune evasion	Immunohistochemistry (IHC) with various cut-offs (1%, 5%, 10%, 50%)	Higher expression associated with worse overall survival in early NSCLC; predictive of benefit from immune checkpoint inhibitors	Imprecise relationship with therapeutic benefit; assay variability; tumor heterogeneity (spatial and temporal); some PD-L1 negative patients still benefit
TMB [43,200]	Total number of somatic mutations in a tumor; higher TMB often correlates with more neoantigens	NGS-based comprehensive genomic profiling	Potential predictor of immunotherapy response; may indicate benefit even in PD-L1 negative cases	Requires further validation and standardization across different cancer types and assays
MSI [43,201]	Genomic instability due to defects in DNA mismatch repair	NGS-based comprehensive genomic profiling	Predicts response to immune checkpoint inhibitors in certain tumor types (e.g., colorectal cancer)	Requires further standardization and understanding of its full predictive value in various cancers
Genetic alterations [159,202] (e.g., KRAS co-mutations)	Influence tumor immune resistance or sensitivity to specific therapies	NGS-based genomic profiling	May indicate resistance to single-agent immunotherapy, suggesting need for combination therapies (e.g., *KRAS/SDK11* or *KRAS/KEAP1* co-mutations)	Complex interplay of multiple mutations; requires comprehensive genomic analysis for detection and interpretation
Immune cell infiltrates [43,199]	Composition and density of immune cells within the tumor microenvironment	Immunohistochemistry, flow cytometry, spatial transcriptomics	May indicate a “hot” tumor more likely to respond to immunotherapy	Challenging to quantify and standardize; dynamic nature of immune microenvironment
Circulating biomarkers (e.g., ctDNA) [145,147]	Tumor-derived molecules in blood reflecting real-time tumor evolution	NGS-based liquid biopsy	Potential for early detection of resistance, monitoring response, and novel biomarker discovery	Low analyte concentration in early stages; challenges in purification and isolation
